# Strengthening breastfeeding counseling through a two-phase training and implementation intervention in Ecuador’s local healthcare system

**DOI:** 10.1186/s13006-026-00866-1

**Published:** 2026-06-19

**Authors:** Fadya Orozco, María Elisa Herrera-Fontana, Stephanie Montenegro, Fabián Muñoz, Johanna Molina, Alicia Chicaiza, Alexis J. Handal

**Affiliations:** 1 Centro de Transferencia de Tecnología CTT-USFQ, Universidad San Francisco de Quito USFQ, Quito, Ecuador; 2 Fundación Tejiendo Maternidades e Infancias - TEMI, Quito, Ecuador; 3 Universidad San Francisco de Quito USFQ, Quito, Ecuador; 4 Visor Análisis Estadístico Cia. Ltda., Quito, Ecuador; 5 DP17-Oficina Técnica 17-OT 10 Cayambe – PedroMoncayo, Cayambe, Ecuador; 6 Department of Epidemiology, University of Michigan School of Public Health, Ann Arbor, Michigan, USA

**Keywords:** Breastfeeding, Training, Healthcare professionals, Breastfeeding counseling, Educational intervention

## Abstract

**Background:**

Breastfeeding counseling is a key intervention to promote optimal infant feeding, particularly when delivered individually and in person. Its effectiveness depends on the quality of healthcare training, which remains variable. In Ecuador, public services primarily rely on group-based strategies with limited access to individualized support, reinforcing inequalities given the high cost of private services. This study describes an intervention co-developed with the local health system to strengthen provider capacity and implement an individualized counseling model in a rural setting.

**Methods:**

This practice-based case study was conducted in two phases between 2023 and 2024. In Phase I, 32 primary-level healthcare professionals participated in an 80-hour training program combining theoretical and practical components focused on technical skills, communication, and supportive counseling. Focus group discussions informed curriculum adjustments and explored changes in perceptions and competencies. In Phase II, a breastfeeding counseling room was established and staffed by ten trained healthcare professionals. Standardized digital records were used to document user characteristics and consultation topics. Data were analyzed descriptively using Stata and thematically using MAXQDA.

**Results:**

Ten of 32 providers (31%) completed the training. A total of 305 counseling sessions were conducted with 247 women, most of whom were lactating (76.5%), aged 18–29 years (68%), and residing in the local catchment area (87.9%). The most frequent consultation topics included breastfeeding technique, nipple pain, perceived low milk supply, and milk expression and storage. Sessions averaged 36 min. Users included adolescents and women with varying levels of maternal experience, highlighting diverse counseling needs. The intervention emphasized individualized, empathetic, and context-sensitive support.

**Conclusions:**

The intervention was feasible within a public primary healthcare setting and addressed key gaps in access to individualized breastfeeding support. It demonstrated the relevance of combining technical and relational competencies in counseling and the importance of integrating such services within existing health systems. These findings provide practical insights for the implementation of similar models in resource-constrained settings.

**Clinical trial number:**

Not applicable.

**Supplementary Information:**

The online version contains supplementary material available at https://doi.org/10.1186/s13006-026-00866-1.

## Background

Breastfeeding counseling is one of the main interventions to promote both the early onset and exclusive practice of breastfeeding and one of the factors the healthcare system may have an impact on [1, 2]. According to the guidelines proposed by UNICEF and the WHO’s Baby Friendly Hospital Initiative (BFHI), one of the critical management procedures is to ensure that healthcare professionals have sufficient knowledge, competencies, and skills to support breastfeeding [3].

In this regard, breastfeeding counseling, conceived as an individualized intervention that combines communication, educational, and technical skills to support informed decision-making by mothers—requires specific and solid training to be effective [4–6]. Its impact is greater when offered personally during the postnatal period and adapted to each woman’s context, as it facilitates early problem resolution and addresses concerns related to infant feeding [1, 2, 5, 7]. Therefore, the quality of counseling is closely linked to the training of the professionals providing it [4, 8, 9].

Despite the existence of international guidelines for training healthcare staff in breastfeeding counseling [6], there is considerable variability in how these trainings are implemented [8, 10]. Approaches differ in format (in-person, virtual, or mixed), content focus, and duration, which can range from 12 to 133 h [8, 10–12]. Additionally, key components such as empathetic communication, active listening, and respect for cultural diversity are not always sufficiently addressed [1, 4]. Evidence suggests that training programs combining theory and practice, particularly those emphasizing practical components, are more effective in strengthening providers’ self-efficacy and improving counseling practices [4, 11–13].

At the same time, healthcare professionals often present heterogeneous levels of knowledge and skills in breastfeeding counseling due to differences in pre-service education and limited opportunities for continuing training, particularly in public healthcare systems [4, 10].

This issue is especially relevant in Ecuador, where recent data show a decline in breastfeeding indicators and ongoing challenges in implementing effective promotion strategies within the public healthcare system. The prevalence of exclusive breastfeeding up to six months decreased from 62% in 2018 to 53% in 2024 [14, 15].

Within the Ecuadorian public healthcare system, breastfeeding promotion is mainly based on community support groups facilitated by primary-level healthcare professionals, who are often trained through theoretical and virtual modalities. These groups provide spaces for sharing experiences among pregnant and breastfeeding women [16]. However, their impact on exclusive breastfeeding is limited compared to individualized counseling interventions delivered within healthcare services [2], although combined strategies may produce synergistic effects [1, 2].

Individual counseling services are also available in the private sector but at costs ranging from 40 to 60 USD per session, representing a significant barrier in a context where the minimum salary is 470 USD. This reflects structural inequalities in access to maternal and child healthcare, particularly affecting women in vulnerable settings [17, 18].

In response, countries such as Chile [19, 20] and Mexico [21] have implemented breastfeeding clinics within public healthcare systems to provide accessible and continuous support. In Mexico, 91% of women received breastfeeding counseling in the public sector compared to 80% in the private sector, demonstrating the feasibility of integrating such services into public institutions [22].

In this context, two major gaps were identified in the provision of public breastfeeding support services: (1) limited access to individualized counseling [16–18], and (2) the absence of institutionalized spaces within the healthcare system to provide continuous and affordable support.

To the best of our knowledge, this is the first initiative of its kind within the Ecuadorian public healthcare system.

This article aims to document and describe an intervention developed in collaboration with the local healthcare system, focused on strengthening healthcare professionals’ competencies and implementing an individualized breastfeeding counseling model in a predominantly rural area of Ecuador. It further analyzes its implementation, lessons learned, and potential applicability in similar contexts.

### Study design

The study followed a practice-based, mixed-methods approach combining qualitative data (focus group discussions), structured observation of participant performance during training, and descriptive analysis of service utilization data. These sources were used in a complementary manner through the integration of findings across data sources, allowing for triangulation and an implementation-focused understanding of the process.

This study was conceived as an implementation-focused documentation process aimed at extracting lessons learned rather than establishing causal effects. Qualitative methods (focus group discussions in Phase I) explored participants’ experiences, knowledge, and attitudes, while quantitative-descriptive analysis of care records in Phase II characterized service utilization.

The intervention consisted of two phases: (1) a theoretical–practical training process for primary-level healthcare professionals to strengthen breastfeeding counseling competencies; and (2) the implementation of a breastfeeding counseling room within a public healthcare unit, providing individualized support to pregnant and breastfeeding women.

The intervention was conducted as part of the knowledge-transfer activities associated with the SEMILLA birth cohort study, implemented in the Cayambe–Pedro Moncayo area, Ecuador [23], which previously identified gaps in breastfeeding confidence, suboptimal exclusive breastfeeding practices, and high levels of child malnutrition in the study population [15, 24].

Ethical approval was granted by the Ethical Committee for Research in Humans of Universidad San Francisco de Quito (CEISH, ID 2017-177IN; approved February 8, 2018) within the framework of the SEMILLA study. The District Health Division 17D10 (Cayambe–Pedro Moncayo) also authorized the implementation of the intervention. All participants in the focus groups provided oral informed consent.

### Study area and participants

The intervention took place in Health District 17D10 (Cayambe–Pedro Moncayo), which includes one secondary-level hospital and 15 primary care centers. The district serves a largely rural population with diverse socioeconomic and ethnic characteristics [25, 26].

Participants were healthcare professionals involved in maternal and child care, including obstetricians, physicians, and primary-level care technicians (TAPS). A total of 32 professionals participated in Phase I. Participants were selected through convenience sampling and identified by district health authorities based on their routine roles in providing care to pregnant and breastfeeding women.

Two district managers participated both as trainees and as facilitators of the implementation process.

#### Phase I: Breastfeeding counselor training

The training was conducted by three facilitators with expertise in nutrition, medicine, and public health. It consisted of an 80-hour program combining theoretical sessions and supervised practical training over eight months.

The curriculum was structured around three core areas: (1) empowerment-oriented health education, (2) communication in healthcare, and (3) technical aspects of breastfeeding. These components were integrated within a practice-based pedagogical model combining theory and experiential learning, including role plays, case simulations, and supervised counseling practice [4, 27–29].

The training approach was informed by a liberating education perspective grounded in Paulo Freire’s critical pedagogy, emphasizing dialogue, reflection, and the recognition of women’s lived experiences as central to the counseling process [30]. The articulation of these components throughout the training is summarized in Table 1.

The same curriculum was delivered to all participants without initial adaptation by professional profile. Differences in baseline knowledge and performance emerged during the process and are reflected in the results.

Training materials were developed for this intervention based on international guidelines and facilitators’ experience and may be shared upon request.

**Table 1:** Core topics and themes treated in the breastfeeding counseling staff training. Cayambe-Pedro Moncayo Health District

Curricular axes	Main topics
Empowerment	- Foundations of health education - Liberating education for breastfeeding support - Concept of power and empowerment in health promotion practice
Health Communication	- Active listening - Verbal and non-verbal communication - Assertiveness and empathy - Mother-centered care model - Communication tailored to the mother’s profile (age, culture, educational level) - Use of plain and clear language - Building trust between the mother and the counselor - Facilitating the mother’s participation in decision making
Specialized technical content	- Anatomy and physiology of lactation - Infant nutrition - Milk production: barriers and facilitators - Breastfeeding techniques - Common breastfeeding challenges: pain, engorgement, poor weight gain, and swallowing difficulties - Breast milk expression techniques - Establishing a home-based milk bank - Breastfeeding in special situations: prematurity, low birthweight, HIV, tuberculosis, and other conditions - Kangaroo Mother Care for mothers and fathers

The training combined theoretical and practical sessions delivered in both classroom and clinical settings. Theoretical sessions included a mix of explanatory and interactive methods, such as theoretical reviews, role plays, case simulations, practical demonstrations, and guided exercises, accompanied by structured feedback [4, 27–29].

Practical sessions involved supervised counseling practice with pregnant and breastfeeding women in healthcare facilities. Participants worked in small groups under facilitator guidance, taking turns conducting counseling sessions while peers observed. This approach aimed to strengthen confidence and support the development of decision-making skills in real-world contexts. Oral consent was obtained from all participating mothers prior to these sessions.

#### Assessment and training completion

Assessment was conducted using a competency-based approach integrated into the training process, focusing on observed performance rather than standardized or externally validated instruments.

Baseline knowledge was explored through an initial structured questionnaire used to identify general knowledge gaps. Participants’ competencies were assessed through structured observation of counseling performance during role plays and supervised practice, using a predefined evaluation framework (Table 2), which integrated technical knowledge, communication skills, and relational competencies such as empathy, active listening, and respect for the mother’s context. The framework was developed specifically for this training program based on the competencies emphasized throughout the curriculum. Assessments were conducted by the same three facilitators responsible for delivering the training, all of whom had expertise in nutrition, medicine, and public health.

Attitudinal dimensions were not measured as an isolated construct but were assessed indirectly through observable behaviors during counseling interactions and complemented by qualitative feedback. A final focus group discussion was conducted to explore participants’ perceptions of the training program and perceived changes in their counseling approach.

Formative assessments were conducted in 20 of the 25 training sessions to provide continuous feedback on participants’ strengths and areas for improvement; these assessments were not graded. The first two sessions did not include evaluation. Three sessions were designated for summative evaluation, during which participants’ performance was formally assessed using the same predefined criteria.

Participants were required to attend at least 70% of the training sessions. Failure to meet this threshold, or two consecutive absences, resulted in exclusion from the course. Final performance was determined based on summative assessment results in combination with attendance compliance.

Participants who met both attendance and performance criteria were considered to have successfully completed the training program. Successful completion of the training program did not constitute a formal or externally accredited qualification. The evaluation criteria used to assess participants’ competencies are detailed in Table 2.

**Table 2:** Integral assessment: Breastfeeding counseling and practice evaluation

Criterion	Description	Observable indicators	Success scale
1. Welcome and therapeutic bond	Starts interaction showing respect, openness and creating trust.	- Gives a warm welcome. - Explains the session’s purpose. - Shows respect for dignity and privacy.	1 = Not achieved 2 = Partially achieved 3 = Adequate 4 = Excellent
2. Active listening and context exploration	Understands the mother, listening actively; acknowledges her personal, social and cultural circumstances.	- Maintains eye contact and open posture. - Identifies barriers and facilitators. - Considers age, culture and level of education.	1 = Not achieved 2 = Partially achieved 3 = Adequate 4 = Excellent
3. Clear and adaptive communication	Explains topics in simple language, adjusted to the mother’s profile.	- Avoids unnecessary technicisms. - Relates information to previous experiences. - Checks understanding through questions or examples.	1 = Not achieved 2 = Partially achieved 3 = Adequate 4 = Excellent
4. Practical demonstration and application	Demonstrates and practically guides the breastfeeding issue or technique.	- Carries out demonstration step by step. - Allows mother to practice and seek feedback. - Uses practical resources from the environment.	1 = Not achieved 2 = Partially achieved 3 = Adequate 4 = Excellent
5. Breastfeeding technique	Assesses key technical elements during feeding.	- Comfortable posture of mother. - Alignment and closeness of baby. - Good mouth-breast latching. - Recognizes effective sucking and adequate feeding.	1 = Not achieved 2 = Partially achieved 3 = Adequate 4 = Excellent
6. Promotion of self-confidence and empowerment	Reinforces mother’s security and furthers her decision-making autonomy.	- Validates mother’s achievements and capacities. - Motivates to identify own solutions (liberating education). - Promotes protagonism and confidence (empowerment).	1 = Not achieved 2 = Partially achieved 3 = Adequate 4 = Excellent
7. Proposed action and shared decision-making	Provides recommendations, builds agreements with mother and family.	- Presents practical alternatives. - Involves family/caretakers when adequate. - Furthers shared decision-making.	1 = Not achieved 2 = Partially achieved 3 = Adequate 4 = Excellent
8. Motivating closure and continuity	Ends consolidating the lessons learned and motivates for action.	- Summarizes key points. - Reinforces confidence and motivation. - Offers future support and follow-up.	1 = Not achieved 2 = Partially achieved 3 = Adequate 4 = Excellent

Scores were calculated by summing the criteria described in Table 2, with a maximum of 32 points, and subsequently converted to a 1–10 scale. A minimum score of 7/10 was required to successfully complete the training.

#### Phase II Implementation of the breastfeeding counseling room

The breastfeeding counseling room was implemented within a primary-level healthcare facility in the Cayambe–Pedro Moncayo Health District, following general guidelines from previous regional experiences [19, 20], while adapting operational and organizational aspects to the local context. Prior to this intervention, no formal individualized breastfeeding counseling service existed within the public healthcare system in the study area; therefore, no baseline utilization data were available.

The implementation involved the identification and adaptation of a physical space within an existing healthcare facility, selected based on its capacity to provide care to pregnant and breastfeeding women, availability of post-partum services, and conditions ensuring privacy and functional integration within routine care.

Standardized procedures and service organization were developed prior to implementation through a collaborative process involving the research team and district health authorities. These procedures were adapted to local service capacity and workflow to ensure feasibility and integration into routine healthcare activities.

The counseling service was integrated into routine care and operated with trained healthcare professionals who had completed the training process. User recruitment was conducted through referrals from routine services, including prenatal care, post-partum care, child health visits, and vaccination services, as well as through direct invitation within the facility.

A standardized counseling record and care guide, developed specifically for the intervention, guided the counseling process. The guide structured the sequence of the consultation, including user reception, collection of sociodemographic and clinical information, assessment of breastfeeding practices, provision of individualized recommendations, and follow-up planning. Consultation time was flexible to allow comprehensive and context-sensitive care. The counseling record and care guide used during implementation are provided as Supplementary File 1.

**Image 1:**
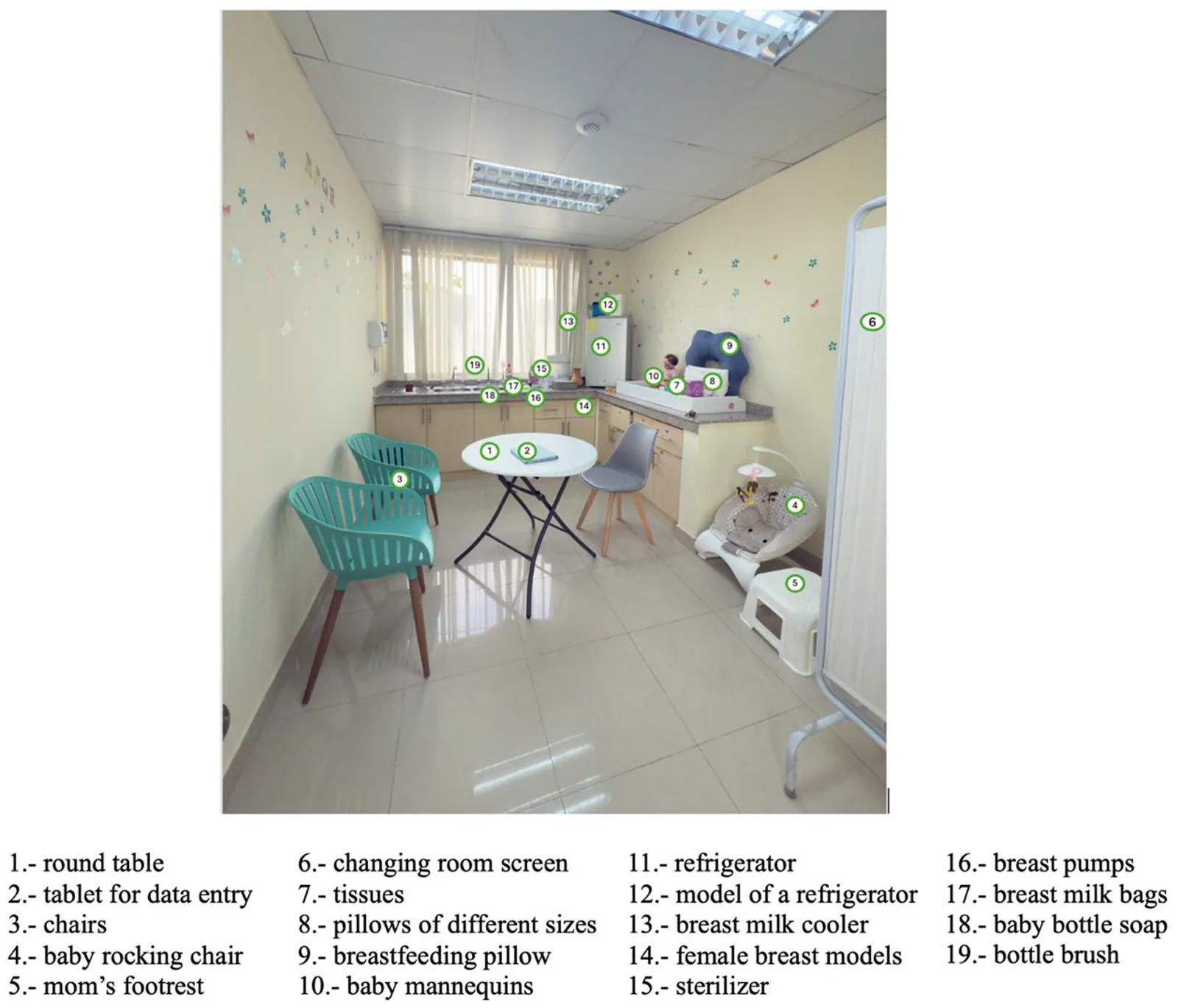
Breastfeeding counseling room at the Type C Healthcare Center in Tabacundo

The counseling room was equipped with basic materials and visual aids adapted to the local context to support counseling activities and facilitate communication with users. The main components of the counseling space are shown in Image 1.

To support service continuity, the intervention was formally incorporated into the district health system, with recognition of counseling activities as part of providers’ institutional responsibilities and the establishment of standardized procedures and resources. This institutional integration was further strengthened by the progressive transfer of operational supervision to the district technical team, reflecting the consolidation of the service within routine care and its capacity to function autonomously over time.

#### Monitoring

Monitoring activities were implemented throughout the intervention to document the process, identify necessary adjustments, and obtain feedback on its implementation, without aiming to conduct an impact or effectiveness evaluation.

During Phase I, three focus group discussions were conducted at different stages of the training process. Two exploratory focus groups were held during week 7 of the course to identify technical challenges and perceived barriers to effective breastfeeding, as reported by healthcare providers. These findings informed subsequent adjustments to the training process. A final focus group was conducted with participants who completed the training to explore their perceptions of the experience and its relevance to their professional practice.

During Phase II, service monitoring was conducted through institutional recording processes integrated into the district health system. Information was initially collected through standardized clinical records, including number of users attended, sociodemographic characteristics, and reasons for consultation. These records were subsequently digitized into a web-based application to facilitate data standardization and systematization. Monitoring of these records was conducted over the implementation period (May–December 2024), allowing for the documentation of service utilization and operational functioning of the counseling model and its practical relevance. Oversight of implementation activities was carried out jointly by the research team and the district technical team, who monitored service operation, adherence to established protocols, and integration within routine healthcare services.

### Analysis

Qualitative data from Phase I focus group discussions were transcribed and analyzed using MAXQDA 24 (VERBI Software GmbH, Berlin). An initial full reading of the transcripts was conducted to familiarize with the data and identify preliminary patterns. An **inductive thematic analysis** was conducted, whereby two authors (FO and FM) independently coded the material and grouped codes into thematic categories based on conceptual similarities that emerged from the data. Categories were reviewed and refined through consensus to ensure coherence and distinctiveness. Representative excerpts were selected to illustrate key themes.

Quantitative data from Phase II were organized from digital records into an Excel database and analyzed using STATA/SE 18.0 through descriptive statistics. Results are presented as frequencies and percentages.

Variables included characteristics of the consultations (number and duration), as well as users’ sociodemographic information, including age, education level, employment status, and place of residence. Reasons for consultation were recorded as open text and subsequently coded into analytical categories using MAXQDA 24.

### Results of the intervention

The results of the intervention are presented focusing on key implementation aspects and lessons learned throughout the process.

### Features of the participants

The group of participants was predominantly female (24 out of 32), consistent with the composition of the maternal and child healthcare workforce in the study setting. Participants represented a range of professional profiles within primary healthcare, including obstetricians, physicians, primary-level care technicians (TAPS), and nutritionists.

Participants also showed wide variability in age and years of professional experience. Ages ranged from 26 to 58 years, while years of experience ranged from 1 to 21 years, reflecting the heterogeneity of the primary healthcare workforce involved in the training process.

Table 3 presents the participants’ occupations at the beginning and at the end of the training, showing the professional profiles at baseline and among those who completed the program.

**Table 3:** Professional profiles of participants at enrollment and among those completing the breastfeeding counselor training

Profession/ working area	Beginning (*n*, %)	Approvals (*n*, %)
Primary-level care technicians (TAPS)	15 (46.8%)	1 (10%)
Obstetricians	12 (37.5%)	5 (50%)
Physicians	3 (9.4%)	3 (30%)
Nutritionists	2 (6.3%)	1 (10%)
**TOTAL**	32 (100%)	10 (100%)

At the end of the training, there was a marked decrease in the representation of primary-level healthcare technicians, while the proportion of obstetricians and physicians increased. These changes reflect a shift in the group’s professional composition, which may suggest that participants with university-level training were more likely to remain in the program and successfully complete it.

In total, 32 participants enrolled, of whom 10 (31%) successfully completed the breastfeeding counseling training. Of these, eight were subsequently involved in the breastfeeding counseling room in Phase II, seven of whom were female.

#### Reasons for not completing the course

Eight participants (25% of the total sample, *n* = 32) were discontinued due to exceeding the non-attendance limit, while fourteen (44%) did not meet the minimum performance required to successfully complete the course. In total, ten participants (31%) met both attendance and performance criteria and were considered to have successfully completed the training.

Among those who successfully completed the training, performance levels were consistently high, with a mean final score of 8.54 (SD = 0.68) and values ranging from 7.56 to 9.79, indicating a strong and relatively homogeneous level of competency acquisition.

In contrast, among participants who did not meet the required performance level (*n* = 14), the main difficulties were concentrated in counselling competencies related to empowering and motivating mothers, breastfeeding technique, and shared decision making. Specifically, lower performance was observed in the domains of promotion of self-confidence and empowerment (6/14), breastfeeding technique (4/14), and proposed action and shared decision making (3/14), with fewer participants showing difficulties in clear and adaptive communication (1/14).

The most frequent difficulties were therefore associated with supporting mothers’ autonomy, fostering confidence, and translating technical knowledge into practical, context-appropriate recommendations.

Overall, while attendance influenced completion, meeting the course’s competency standards represented the main challenge for participants.

### Results of the focus groups conducted during week 7 of the course (Phase I)

#### Barriers for effective breastfeeding

The findings from the first focus group with healthcare providers, collected in Phase I, identified barriers to effective breastfeeding that informed the design of the counseling intervention. These barriers included both practical and knowledge-related challenges affecting breastfeeding counseling and support (Table 4), as well as personal, social, and contextual factors influencing breastfeeding practices (Table 5).

At the level of breastfeeding practices, knowledge limitations were identified among both mothers and some healthcare professionals, as well as difficulties in the application of appropriate techniques and in managing common situations during breastfeeding. These challenges include both technical competencies among health professionals and barriers related to users’ health literacy and communication processes between providers and mothers (see Table 4).

While several of these challenges relate to technical aspects of breastfeeding (e.g., positioning, latch, and milk management), others reflect mothers’ concerns, uncertainties, and contextual constraints, such as perceived milk insufficiency, pain, and the need to reconcile breastfeeding with returning to work.

On the other hand, personal, social, and contextual factors highlight the influence of deeply rooted beliefs, gaps in prenatal support, communication barriers during care, and conditions of particular vulnerability in certain groups, such as adolescent mothers (see Table 5).

Overall, these findings show that breastfeeding challenges extend beyond the clinical domain, encompassing social and relational dimensions that directly influence its practice.

**Table 4:** Breastfeeding counseling challenges identified by healthcare providers during the first focus group of Phase I

Challenge	Synthesis of findings	Illustrative quote
Lacking knowledge of breastfeeding techniques	Many mothers (and some professionals) do not know how to position the baby or ensure an adequate latch.	*“(…) there is lack of knowledge as to how you breastfeed (…).”*
Common breastfeeding concerns reported by mothers	Three central worries: amount of milk, pain/discomfort in nipples, and sustainability returning to work	*“(…) of course*,* ‘cause they say*,* I mean*,* I’m going to work (*…) *and how do I take ‘em out there (…)?”*
Lacking knowledge about milk extraction and conservation	The mothers (and some professionals) do not know the right techniques of milk extraction and storage.	*“(…) there are many things*,* we as healthcare professionals do not know about milk extraction techniques (…).”*

**Table 5:** Personal, social, and contextual factors influencing breastfeeding practices identified by healthcare providers during the first focus group of Phase I

Factor	Description	Illustrative quote
Belief breastfeeding is a natural and instinctive process	Breastfeeding seen as something instinctive not needing preparation or support.	*“(…) the mothers think it is something very natural*,* something they will be able to do ‘normally’ (…).”*
False beliefs regarding the success of breastfeeding	The belief that nipple shape determines breastfeeding success.	*(…) they are worried: their nipples*,* ¿are they going to be right or not (…)”*
Insecurity about milk production	Frequent fear of not producing enough mother milk.	*“(…) they always ask: am I going to have enough milk to satisfy the baby? (…).”*
Adequate time to receive breastfeeding counseling	During pregnancy, there is little interest for issues related to breastfeeding.	*“(…) pregnant women show little interest*,* apparently. They do not ask about breastfeeding. Rather*,* they focus on growth*,* that is the baby’s food (…).”*
Communicational and relational barriers	Limited time for consultation, mothers’ low confidence to ask, healthcare provider’s gender.	*“(…) I think one of the problems there are in counseling is the ladies’ lack of confidence to ask. And*,* besides*,* the time we have is so limited (…).* *“(…) in breastfeeding counseling*,* we’ve had few consultations; the male staff would say they perceive a difference. Women shy away from asking (…).”*
Conditions of vulnerability in adolescent mothers	This population requires a differentiated approach given their social, emotional and physical situation.	“(…) *many of them are alone*,* have no partners (…) so*,* imagine a raped*,* pregnant adolescent*,* what interest might she possibly have in breastfeeding? (…).”*
Early breastfeeding abandonment factors	Lack of education of mother, work and/or household activities, reasons for not breastfeeding	“*(*…) *In my experience (…) the lack of education in pregnant women has an impact (…)”*

#### Curricular adjustments

Based on the findings from the first focus group, the training curriculum was adjusted in both content and methodology to address the technical and relational challenges identified.

In terms of content, new topics were incorporated, including breast milk extraction techniques and home-based milk storage. Although no content was excluded, some topics were reorganized to prioritize their introduction in the early stages of the course, with the aim of strengthening their practical application, particularly those related to common breastfeeding concerns and reasons for consultation.

Methodologically, the practical component of the curriculum was reinforced, with greater emphasis on teaching breastfeeding techniques. In addition, strategies were incorporated to strengthen communication skills among healthcare providers, particularly in relation to empathy and empowerment. These components were addressed through both theoretical and practical sessions using interactive pedagogical tools.

#### Healthcare staff perceptions of the formative experience following the training (Phase I)

In line with the study’s objective of strengthening healthcare professionals’ competencies in breastfeeding counseling, a third focus group was conducted at the end of the training to explore participants’ perceptions of the formative experience. This discussion provided insights into key aspects of the program, particularly those influencing professional practice and interactions with mothers (see Table 6).

**Table 6:** Healthcare staff perceptions of the formative experience after training (Phase I)

Formative experience	Relevance
Theoretical-practical training methodology	*“The course enriched our knowledge in a practical manner*,* since we were not prepared to provide breastfeeding counseling”*
Counseling individualization in order to highlight specific situations	*“(…) this course took into account a lot of things*,* every… mother has different types of problems.”*
Horizontal exchange and active listening	*“(…) we abandoned the ‘we know and they know nothing’ attitude… we actually could learn from them (…) and empower them.”*
Addressing by name (avoiding diminutives and colloquial epithets)	*“…so*,* in order to address them as “mommy” instead of by their name*,* the mothers looked at us as if we were something bigger (…)*,* but we understood that we must treat and address them by their names.”*
Practicing operational empathy to generate trust	*“(…) we got closer to the patient and they begin to relax*,* show more confidence*,* hence gratitude*,* security.”*

Participants reported important changes in their counseling approach. Initially, they described using generalized messages focused on promoting breastfeeding without considering individual contexts. Following the training, they emphasized the need to adapt communication to each woman’s specific situation, address her concerns, and provide more personalized support.

These changes were reflected in improvements in communication and relational practices, including more respectful language, greater attention to individual contexts, and a shift toward more empathetic, person-centered interactions.

### Results of phase II. Implementation of the individualized breastfeeding counseling model

The second phase of the study involved the implementation of a breastfeeding counseling room and the application of the individualized breastfeeding counseling model informed by Phase I findings. Its operation was monitored through the systematic review of digital records collected between May and December 2024, allowing the assessment of both service functionality and the model’s applicability in a real-world context. The analysis focused on counseling dynamics, user profiles, and the main reasons for consultation.

#### Characteristics of the counseling sessions

A total of 305 individual in-person counseling sessions were conducted, involving 247 women. Duration was recorded for 275 sessions, with a mean consultation time of 36 min (SD = 12.2), more than twice the standard duration established in the public healthcare system (15 min).

Most users were women in the breastfeeding period (76.5%), compared to 23.5% of pregnant women, representing a ratio of 3.2:1. This distribution reflects greater utilization of the service during the breastfeeding period, when challenges tend to be more immediate and specific.

Regarding frequency of use, 81.8% of women (*n* = 202) attended a single session, while 18.2% (*n* = 45) attended two or more sessions. Specifically, 13.8% attended twice, 3.6% three times, and 0.8% four times. Although multiple consultations were available, most users accessed the service on a one-time basis.

#### Socio-demographic profile of users

User profiles showed a predominance of young women aged 18–29 years, followed by women aged 30 years or older. Notably, 10.9% were adolescents aged 14–17 years, representing a particularly vulnerable group given their limited experience and reduced access to breastfeeding information, which highlights the need for differentiated counseling approaches (see Table 7).

In terms of education, most users had completed secondary education, while a smaller proportion had higher education. Regarding employment status, most women did not have paid work at the time of attendance, reflecting diverse and often informal employment patterns in the study setting.

In addition, parity data showed that 43.7% of users were primiparous, 41.7% were multiparous, and 9.7% were nulliparous. This variability reflects differences in maternal experience, which may influence breastfeeding practices and counseling needs (see Table 7).

Finally, 87.9% of the women attending the counseling room resided in Pedro Moncayo canton, reinforcing the territorial relevance of the model.

**Table 7:** Socio-demographic characteristics of users attending the breastfeeding counseling room in May-December 2024 (*n* = 247)

Characteristics	Frequency	Percentage
Age in years		
-14–17 -18–29 − 30 or over - no data	27 168 51 1	10.9 68.0 20.7 0.4
Number of children		
- none − 1 − 2 − 3 − 4 − 5 - no data	24 108 64 25 12 2 12	9.7 43.7 26 10.1 4.9 0.8 4.8
Parity		
- Nulliparous -Primiparous - Multiparous - No data	24 108 103 12	9.7 43.7 41.7 4.9
Level of formal education		
- incomplete primary - complete primary - incomplete secondary - complete secondary - incomplete superior - complete superior - no data	2 28 43 113 1 58 2	0.8 11.3 17.4 45.8 0.40 23.5 0.8
Paid work		
- yes - no - no data	71 172 4	28.7 69.7 1.6
Canton of residence		
- Cayambe - Pedro Moncayo - no data	29 217 1	11.7 87.9 0.4

#### Reasons for attending the counseling sessions

**Graph 1:**
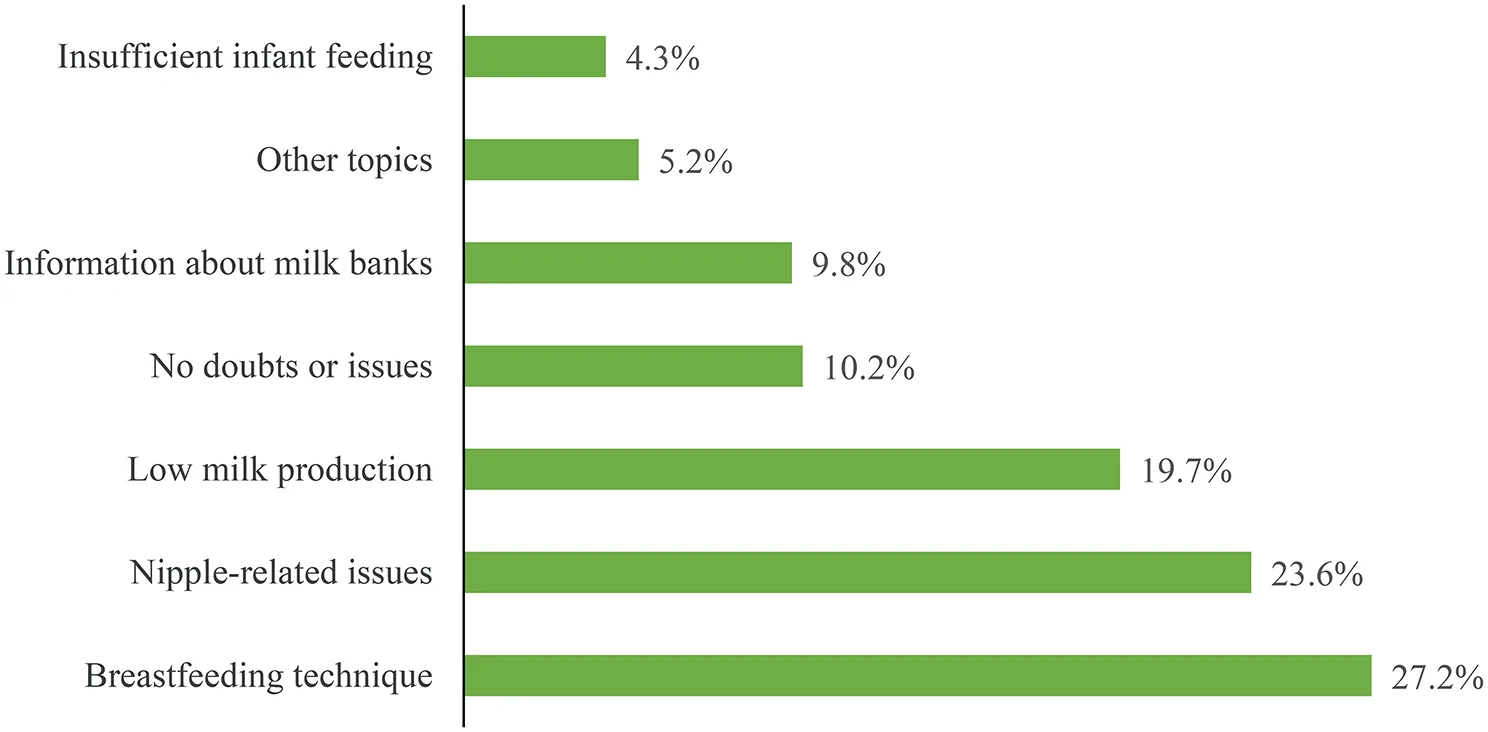
Reasons for attending the breastfeeding counseling room in the Cayambe–Pedro Moncayo District, May–December 2024

Most consultations were related to technical breastfeeding difficulties, primarily breastfeeding technique, nipple-related problems, and perceived low milk production. Overall, these issues accounted for 70% of consultations, representing the largest share of attendance (see Graph 1).

Consultations were also related to planning breastfeeding continuity in the context of returning to work or studies, particularly regarding breast milk extraction and storage. A smaller proportion of women did not express specific concerns at the time of consultation, suggesting that some used the service as a preventive or supportive space.

These findings reflect the need to address both technical challenges and broader support needs across different stages of breastfeeding.

## Discussion

The results of the intervention provide insights into the feasibility and pertinence of integrating an individualized breastfeeding counseling model within the public healthcare system in rural Ecuador. The following sections discuss the main findings in relation to existing literature, practical implications, and future directions.

### Training model

One of the most relevant findings was that only 31% of participants completed the training. Despite a comprehensive methodology integrating theory, role plays, clinical simulations, and field supervision [10–13, 31, 32], barriers such as workload, limited time, and difficulties in aligning training with routine responsibilities affected participation, particularly in primary care settings [33, 34]. These constraints reflect structural conditions of healthcare systems with high demand and limited staffing.

In addition, 44% of participants did not meet the minimum competency requirements, indicating that achieving expected counseling performance was also a significant challenge. Completion patterns showed greater retention among professionals with university-level training, while higher dropout occurred among primary-level technicians (TAPS), highlighting the need for training approaches adapted to diverse professional profiles.

The 80-hour duration should be interpreted in relation to the competencies targeted, including not only technical knowledge but also communication and person-centered care. The training process aimed to support a shift from a predominantly prescriptive model toward a more dialogical, empathetic, and context-sensitive approach to counseling. While extended training facilitated this type of transformation through sustained practice and reflection, it also raises questions about feasibility within routine healthcare settings. Shorter and more flexible models may improve implementation potential.

The training followed a pragmatic, context-adapted approach integrating technical content, counseling skills, and experiential learning. Rather than relying on a fixed pedagogical framework, the model evolved iteratively in response to participants’ needs and local system conditions. Early focus groups contributed to identifying gaps and later served an evaluative function, consistent with implementation-focused research aimed at documenting feasibility rather than causal effects [23].

Institutional support played a critical enabling role. Flexibility in schedules and work organization facilitated both training and service implementation. Such support is a key factor for sustaining innovations in resource-constrained settings [35–37]. The consolidation of a small group of trained professionals also contributed to establishing local technical capacity and potential “breastfeeding champions” within the system [8, 38].

Together, these findings provide relevant insights for the design of training strategies in primary healthcare, highlighting the importance of contextual adaptation, iterative implementation, and institutional commitment.

### Implementation of the breastfeeding counseling room

The establishment of a breastfeeding counseling room represented one of the most tangible outcomes of the intervention. Its implementation required adapting physical space, developing standardized counseling procedures through a collaborative process, organizing service flows, and integrating the service into routine healthcare activities, which is particularly relevant in resource-limited rural contexts.

Unlike countries such as Chile, where breastfeeding clinics are institutionally supported within national programs [20], this experience represents an initial effort within the Ecuadorian public healthcare system, implemented without a formal national regulatory framework but with strong local institutional support. It demonstrates that individualized counseling services can be integrated into primary care settings when supported by trained personnel, institutional commitment, and context-adapted organization.

This experience contributes to the still limited body of evidence in Latin America on how to operationalize and integrate breastfeeding counseling services within public healthcare systems in middle- and low-income countries. Beyond its implementation, the process itself generated practical insights into how such services can be structured, adapted, and sustained within existing system constraints.

The sustained operation of the counseling room, the use of routine monitoring data, and the observed demand for services suggest that such interventions can be locally embedded and operationally viable. These findings highlight the importance not only of institutional support and trained personnel, but also of iterative, context-sensitive implementation processes as key components for strengthening maternal and child health strategies.

### Features of counseling sessions and implications for the care model

The counseling approach was characterized by longer and more personalized interactions compared to standard public healthcare consultations. This is consistent with evidence highlighting the importance of sufficient time, active listening, and emotional support for effective breastfeeding counseling [9, 10, 39], particularly in models centered on care experience and relational engagement. These findings suggest the need to reconsider consultation timeframes when aiming to deliver person-centered maternal and child care.

Service utilization patterns indicate that counseling addresses a significant need, particularly during the postpartum period, when mothers face both practical and emotional challenges requiring tailored support [40]. However, the low proportion of repeat visits suggests barriers to continuity of care. As reported in the literature, factors such as caregiving responsibilities and time constraints may limit the ability to attend follow-up sessions [18].

These findings highlight the importance of incorporating proactive follow-up strategies, such as scheduled appointments, reminders, or outreach mechanisms, to strengthen continuity and enhance the effectiveness of counseling interventions [3, 41].

### User profiles and vulnerability

User profiles reflect multiple, intersecting dimensions of vulnerability that are relevant for the design of context-sensitive interventions. Adolescents represented 11% of users, consistent with national trends [42], and face additional barriers such as limited support networks, social stigma, and, in some cases, situations of abandonment or violence. These conditions, shaped by broader social and relational contexts, may hinder breastfeeding continuity and reinforce the need for differentiated and context-aware support approaches.

In terms of education, women with secondary or higher education were more likely to access services, consistent with patterns described in the literature [42]. While this may facilitate engagement with counseling, it also reflects underlying inequalities in access to information and healthcare services. This pattern raises concerns about the potential exclusion of women with lower educational levels and highlights the importance of strengthening integration with routine maternal and child health services to broaden access among more vulnerable populations [17, 18].

Differences by parity further illustrate how maternal experience shapes counseling needs. In this study, 43.7% of users were primiparous and 41.7% were multiparous. First-time mothers may require greater technical and emotional support, while multiparous women may face additional constraints related to caregiving responsibilities and time limitations [43]. These findings underscore the importance of adapting counseling to different maternal trajectories and life circumstances.

Geographic proximity also influenced access, with most users residing near the service location. This pattern reflects not only logistical factors but also territorial inequalities in service availability, highlighting the importance of spatial planning that considers both equity and operational feasibility in service delivery [18].

### Demand and pertinence

The main reasons for consultation were practical challenges such as positioning, pain, perceived low milk production, and milk extraction or storage. These issues are widely documented and associated with early breastfeeding discontinuation [10, 36, 44–49], particularly among women facing structural and contextual barriers.

Across both phases of the intervention, findings showed a clear convergence between the challenges identified during training and the reasons for consultation in practice. Through triangulation of qualitative insights from focus group discussions, observed performance, and service utilization data, the study provides a coherent picture of how these needs manifest in real care settings.

Initial perceptions among providers tended to underestimate these challenges, reflecting the widespread belief that breastfeeding is a natural and instinctive process. The training process contributed to reframing this perspective, highlighting the need for a more technical, personalized, and context-sensitive approach to counseling [45], particularly for women with limited experience or in vulnerable conditions.

In this context, the observed demand is closely aligned with the relevance of the counseling model. By combining technical guidance with active listening and emotional support, the approach is better suited to address situations marked by uncertainty, physical discomfort, or lack of confidence, where standardized information alone may be insufficient.

### Limitations

This study was not designed as an impact evaluation, and the absence of a control group limits causal interpretation. However, the mixed-methods approach, including training follow-up, focus group discussions, and service utilization data, allowed for a comprehensive understanding of the implementation process and the perceived value of the intervention within real-world conditions.

The scope of data collected in the counseling records was intentionally limited to variables directly relevant to breastfeeding support (e.g., number of children, education level, and reason for consultation), as the service was designed as a counseling and support space rather than a comprehensive clinical care unit. Therefore, extensive clinical or sociodemographic information typically included in full medical records, such as detailed obstetric history and occupational status, was not systematically collected, limiting the exploration of how these factors may influence breastfeeding practices and service utilization.

The 80-hour training process provided important insights not only into competency development but also into the conditions required for implementation within primary care. In this sense, the challenges encountered, particularly those related to workload and institutional demands, should be interpreted not only as limitations, but as part of the learning generated through the process. These findings point to the need for more flexible and context-adapted training strategies in similar settings.

Finally, the dual role of facilitators as researchers may have influenced participants’ responses. However, the use of multiple sources of information allowed for a more integrated interpretation of findings, identifying consistent patterns across training experiences and service delivery.

Future research should build on these findings through designs incorporating comparison groups, longer-term follow-up, and external observation, in order to further explore effectiveness and sustainability. Despite these limitations, the study provides relevant insights into the feasibility and contextual applicability of individualized breastfeeding counseling in rural primary healthcare settings.

### Policy implications

From a health systems perspective, these findings point to three actionable priorities for strengthening breastfeeding support within primary healthcare.

First, training approaches should be modular and context-adapted, recognizing the heterogeneity of professional backgrounds within primary healthcare teams. Flexible and differentiated training formats, adapted to diverse competencies and learning needs, may be more effective than uniform, standardized programs.

Second, the implementation of individualized counseling services requires institutional support and appropriate workforce organization. This includes staff allocation, integration into routine responsibilities, and sustained commitment from health system actors to ensure that these services can be effectively delivered within existing healthcare structures.

Third, strengthening breastfeeding support requires shifting away from standardized, group-based education models toward individualized counseling approaches. Current strategies, often centered on generic, infant-focused messages, may be insufficient to address the diverse and context-specific needs of women. Policy efforts should therefore prioritize the integration of individualized, person-centered counseling into routine care, ensuring adequate consultation time and emphasizing communication and relational competencies as essential components of maternal and child health services.

## Conclusion

This study demonstrates the feasibility of implementing an individualized breastfeeding counseling model within a rural primary healthcare setting in Ecuador using existing institutional resources. The intervention, structured through the training of healthcare professionals and the establishment of a counseling service, highlights how practice-based approaches can be effectively integrated into routine care.

Findings underscore the importance of combining technical knowledge with personalized, context-sensitive support, particularly for vulnerable groups such as adolescents and women with limited maternal experience. The demand for counseling focused on practical and emotional challenges further reinforces the need for individualized approaches to address breastfeeding during the postpartum period.

The integration of counseling into existing services, supported by locally trained personnel and institutional commitment, suggests a viable pathway for strengthening breastfeeding support in resource-constrained settings. More broadly, these findings highlight the need to move beyond standardized approaches and to incorporate individualized, person-centered counseling as a core component of maternal and child healthcare.

Future research should explore the effectiveness and sustainability of such models through more rigorous designs and longer-term evaluation.

## Supplementary Information

Below is the link to the electronic supplementary material.


Supplementary Material 1


## Data Availability

The datasets generated and/or analyzed during this study include transcripts of focus group discussions and anonymized counseling records collected through standardized clinical forms. Due to privacy and ethical considerations, these data are not publicly available but may be available from the corresponding author upon reasonable request.
