# Supplementary material for: Strengthening breastfeeding counseling through a two-phase training and implementation intervention in Ecuador’s local healthcare system
Source: Int Breastfeed J. 2026 Jun 19;21:82. doi: 10.1186/s13006-026-00866-1 (PMC13536587; doi:10.1186/s13006-026-00866-1)
Supplement: Supplementary file 1 [file 13006_2026_866_MOESM1_ESM.pdf]

SALA DE ASESORIA EN LACTANCIA MATERNA

Ficha de atención

Fecha de atención: ________/________/________ **Hora de inicio:** ____:____ **Hora de fin:** ____:____

Nombre de la asesora: ____

Usuaria lactante ☐ **Usuaria gestante** ☐

1. ***Bienvenida cálida*** *a la usuaria*

2. *Por favor, me recuerda* ***¿cuál es su nombre?***

   Nombres y apellidos de la usuaria:____

3. *¿Cómo le gustaria que le diga? (nombre) ó Sra (nombre)*
4. *¿Cuántos años tiene?*

   Edad: ______ años

5. *¿Qué nivel de estudio alcanzó?*

   ____

6. *¿Trabaja* ***por un ingreso económico****? (Señalar con una X la respuesta correspondiente)*

   Si ☐ *¿Dónde?* ____
   No ☐

7. *¿Dónde vive? (Señalar con una X el cantón y la parroquia correspondiente)*

   Cayambe ☐
   - *Ascázubi* ☐
   - *Cusubamba* ☐
   - *Otón* ☐
   - *Cangahua* ☐
   - *Cayambe* ☐
   - *Juan Montalvo* ☐
   - *Ayora* ☐
   - *Olmedo* ☐
   - *Pesillo* ☐

   Pedro Moncayo ☐
   - *Malchingui* ☐
   - *Tocachi* ☐
   - *La Esperanza* ☐
   - *Tabacundo* ☐
   - *Tupigachi* ☐

   Otro: ☐ *especificar:* ______________________

8. ¿Con quién vive? *(Señalar con una X la respuesta correspondiente)*

   Padres ☐
   Pareja ☐
   Hijos ☐
   Otros familiares ☐ Especificar:____
   Otros no familiares ☐ Especificar: ____

SALA DE ASESORIA EN LACTANCIA MATERNA

Ficha de atención

9. ¿Tiene alguna enfermedad? *(Señalar con una X la respuesta correspondiente)*

Si ☐ *¿Cuál?* ____

No ☐

10. ¿Recibe atención por algún **servicio de salud**, como por ejemplo: salud mental?
(Señalar con una X la respuesta correspondiente)

Si ☐ *¿Cuál?* ____

No ☐

11. ¿Toma algun **medicamento**? *(Señalar con una X la respuesta correspondiente)*

Si ☐ *¿Cuál?* ____

No ☐

12. ¿**Cuántos hijos** tiene? *(Señalar con una X el número de hijos reportado por la usuaria)*

| 1 | 2 | 3 | 4 | 5 | 6 | 7 | 8 | 9 | 10 |

13. Antecedentes reproductivos *(Señalar con una X el número reportado por la usuaria)*

¿Cuántas veces ha estado **embarazada**? | 1 | 2 | 3 | 4 | 5 | 6 | 7 | 8 | 9 | 10 |

¿Cuántas veces ha dado a luz por **parto vaginal**? | 1 | 2 | 3 | 4 | 5 | 6 | 7 | 8 | 9 | 10 |

¿Cuántas veces ha dado a luz por **cesárea**? | 1 | 2 | 3 | 4 | 5 | 6 | 7 | 8 | 9 | 10 |

¿Ha tenido **abortos**?

Si ☐ *¿Cuántos?* | 1 | 2 | 3 | 4 | 5 | 6 | 7 | 8 | 9 | 10 |

No ☐

14. ¿Ha acudido antes a esta sala de asesoria de lactancia materna? *(Señalar con una X la respuesta correspondiente)*

Si ☐

No, es la primera vez ☐

15. ¿Quién le refirió a este espacio?

Nombre de la persona:______________________________

Unidad de salud/ Institución a la que pertenece: ______________________________

16. ¿Cuál es el motivo de su consulta?

____

En caso de que la usuaria reporte que no tiene dudas sobre la lactancia, dígale cálidamente que por favor le enseñe cómo le da de lactar a su bebé. En el caso de usuarias embarazadas ofrecer la muñeca y el seno de silicona. Observar la técnica de lactancia y reforzarla.

SALA DE ASESORIA EN LACTANCIA MATERNA

Ficha de atención

17. *Escribir datos importante sobre el contexto relatado por la Sra*

____
____
____

18. ***Examen físico de la mama***

*****_**Guía de exploración**_

Mama Derecha

MAMA	***PEZÓN***
Número	***Número***
Forma	***Forma***
Tamaño	***Tamaño***
Signos de inflamación:	***Signos de inflamación:***
Eritema (color rojo):	*Eritema (color rojo):*
Dolor a la palpación:	*Dolor a la palpación:*
Calor a la palpación: Consistencia/Congestión:	*Calor a la palpación:*
Lesiones	***Lesiones***
Cicatrices previas	***Cicatrices previas***
Otros	***Otros***

Mama Izquierda

MAMA	***PEZÓN***
Número	***Número***
Forma	***Forma***
Tamaño	***Tamaño***
Signos de inflamación:	***Signos de inflamación:***
Eritema (color rojo):	*Eritema (color rojo):*
Dolor a la palpación:	*Dolor a la palpación:*
Calor a la palpación: Consistencia/Congestión:	*Calor a la palpación:*
Lesiones	***Lesiones***
Cicatrices previas	***Cicatrices previas***
Otros	***Otros***

19. *Examen de la técnica de lactancia*

******Guía de exploración***

a. Postura de la madre
   - Posición cómoda para dar de lactar según el contexto de la usuaria
   - Ofrece el seno con la mano en forma de “C”
   - Pezón debe apuntar al paladar del bebé
b. Posición del bebé
   - Alineación al cuerpo de la madre
   - Mentón en contacto con el pecho
   - Nariz libre

     *Explicar que para sacar al bebé del pecho, lo mejor es introducir el dedo meñique de la madre en la comisura del labio, para romper el vacio creado al momento de la succión
c. Agarre
   - Mentón del bebé toca su pecho
   - Boca abierta y agarre de toda la areola
   - Labios hacia afuera (evertidos)
   - Mejillas redondas/ llenas al succionar
   - Madre sin dolor
d. Succión
   - Movimientos lentos y suaves de la mandíbula
   - Movimientos coordinados con respiración y deglución
   - Sonido de deglución

SALA DE ASESORIA EN LACTANCIA MATERNA

Ficha de atención

20. *Examen físico del bebé (Señalar con una X la respuesta correspondiente)*

Labio leporino ☐
Paladar hendido ☐
Otro ☐ *Especificar:* ______________________

21. *Acciones y recomendaciones*

Tema de asesoría:

☐ Anatomía de la mama
☐ Lactogénesis
☐ Técnica de amamantamiento
☐ Posturas de lactancia
☐ Banco de leche
☐ Método canguro
☐ Otro Especificar:________________________________

Acciones: ____

____

____

____

Recomendaciones:

____

____

____

____

Agendamiento para una nueva visita

Fecha de la próxima visita: ______________________

Número de contacto:__________________________
